# Key recommendations for primary care from the 2022 Global Initiative for Asthma (GINA) update

**DOI:** 10.1038/s41533-023-00330-1

**Published:** 2023-02-08

**Authors:** Mark L. Levy, Leonard B. Bacharier, Eric Bateman, Louis-Philippe Boulet, Chris Brightling, Roland Buhl, Guy Brusselle, Alvaro A. Cruz, Jeffrey M. Drazen, Liesbeth Duijts, Louise Fleming, Hiromasa Inoue, Fanny W. S. Ko, Jerry A. Krishnan, Kevin Mortimer, Paulo M. Pitrez, Aziz Sheikh, Arzu Yorgancıoğlu, Helen K. Reddel

**Affiliations:** 1 Locum General Practitioner, London, UK; 2 Department of Pediatrics, Vanderbilt University Medical Center, Nashville, TN USA; 3 Department of Medicine, University of Cape Town, Cape Town, South Africa; 4 Québec Heart and Lung Institute, Université Laval, Québec City, QC Canada; 5 Institute for Lung Health, Leicester NIHR BRC, University of Leicester, Leicester, UK; 6 Pulmonary Department, Mainz University Hospital, Mainz, Germany; 7 Department of Respiratory Medicine, Ghent University Hospital, Ghent, Belgium; 8 Departments of Epidemiology and Respiratory Medicine, Erasmus Medical Center, University Medical Center Rotterdam, Rotterdam, The Netherlands; 9 ProAR Foundation and Federal University of Bahia, Salvador, Bahia Brazil; 10 Brigham and Women’s Hospital and Department of Medicine, Harvard Medical School, Boston, MA USA; 11 Divisions of Respiratory Medicine and Allergology and Neonatology, Department of Pediatrics, Erasmus MC, University Medical Center Rotterdam, Rotterdam, The Netherlands; 12 National Heart and Lung Institute, Imperial College, London, UK; 13 Department of Pulmonary Medicine, Kagoshima University Graduate School of Medical and Dental Sciences, Kagoshima, Japan; 14 Department of Medicine and Therapeutics, The Chinese University of Hong Kong, Hong Kong, China; 15 Breathe Chicago Center, University of Illinois Chicago, Chicago, IL USA; 16 Liverpool University Hospitals NHS Foundation Trust, Liverpool, UK; 17 University of Cambridge, Cambridge, UK; 18 Department of Paediatrics and Child Health, College of Health Sciences, School of Clinical Medicine, University of KwaZulu-Natal, Durban, South Africa; 19 Hospital Santa Casa de Porto Alegre, Porto Alegre, Brazil; 20 Department of Primary Care Research & Development, Usher Institute, University of Edinburgh, Edinburgh, UK; 21 Department of Pulmonology, Celal Bayar University, Manisa, Turkey; 22 The Woolcock Institute of Medical Research and The University of Sydney, Sydney, NSW Australia

**Keywords:** Asthma, Therapeutics

## Abstract

The Global Initiative for Asthma (GINA) was established in 1993 by the World Health Organization and the US National Heart Lung and Blood Institute to improve asthma awareness, prevention and management worldwide. GINA develops and publishes evidence-based, annually updated resources for clinicians. GINA guidance is adopted by national asthma guidelines in many countries, adapted to fit local healthcare systems, practices, and resource availability. GINA is independent of industry, funded by the sale and licensing of its materials. This review summarizes key practical guidance for primary care from the 2022 GINA strategy report. It provides guidance on confirming the diagnosis of asthma using spirometry or peak expiratory flow. GINA recommends that all adults, adolescents and most children with asthma should receive inhaled corticosteroid (ICS)-containing therapy to reduce the risk of severe exacerbations, either taken regularly, or (for adults and adolescents with “mild” asthma) as combination ICS–formoterol taken as needed for symptom relief. For patients with moderate–severe asthma, the preferred regimen is maintenance-and-reliever therapy (MART) with ICS–formoterol. Asthma treatment is not “one size fits all”; GINA recommends individualized assessment, adjustment, and review of treatment. As many patients with difficult-to-treat or severe asthma are not referred early for specialist review, we provide updated guidance for primary care on diagnosis, further investigation, optimization and treatment of severe asthma across secondary and tertiary care. While the GINA strategy has global relevance, we recognize that there are special considerations for its adoption in low- and middle-income countries, particularly the current poor access to inhaled medications.

## Introduction

Asthma affects more than a quarter of a billion people worldwide, is the most common chronic condition in childhood, and is responsible for over 1000 deaths a day, of which the majority are preventable^1–4^.

The Global Initiative for Asthma (GINA) was established by the World Health Organization and the US National Heart Lung and Blood institute in 1993 to improve asthma awareness, prevention, and management worldwide. GINA is independent of industry, funded by the sale and licensing of its evidence-based, annually updated reports and figures. The GINA methodology is published on its website (https://ginasthma.org/about-us/methodology).

The GINA report is a global evidence-based strategy that can be adapted for local health systems and local medicine availability. Many countries have their own national asthma guidelines, with many of these based on GINA^5^. However, most national guidelines are updated only infrequently, so they may not reflect current best evidence. In recent years, some countries have conducted partial updates of their asthma guidelines, by undertaking a detailed review of evidence for a limited number of clinical questions, but this process often takes several years. By contrast, the GINA strategy is updated every year based on a twice-yearly cumulative review of new evidence. Hence, even when national asthma guidelines are available, the GINA report may provide a useful resource for clinicians (both primary care and specialists) to be aware of the most recent evidence, and to understand how it can be integrated into holistic asthma care. However, when assessing and treating patients, health professionals are strongly advised to use their own professional judgment, and to take into account local and national regulations and guidelines, and the needs of the individual patient.

While the GINA strategy report is intended to have global relevance, there are particular considerations for asthma management in low- and middle-income countries^6,7^. Of particular concern is the widespread lack of access to affordable diagnostic tools and inhaled medications, which contributes substantially to the heavy burden of asthma mortality and morbidity seen in these countries.

At the most fundamental level, patients in many areas do not have access even to low-dose inhaled corticosteroids (ICS), which are the cornerstone of care for asthma patients of all severity.

GINA collaborates with and strongly supports the call by the International Union against Tuberculosis and Lung Diseases for a World Health Assembly Resolution on universal access to affordable and effective asthma care, as a step towards addressing these needs^8^.

GINA is also a partner organization in a program launched in March 2006 by the World Health Organization (WHO) and the Global Alliance against Chronic Respiratory Diseases (GARD). Through the work of GINA, and in co-operation with GARD and with the International Union Against Tuberculosis and Lung Diseases, substantial progress toward better care for all patients with asthma globally should be achieved in the next decade.

To achieve this, GINA believes that the safest and most effective approach to asthma treatment in adolescents and adults, which also avoids the consequences of starting treatment with short-acting beta_2_ agonists (SABA) alone, depends on access to ICS–formoterol across all asthma severity levels. With budesonide-formoterol now on the WHO essential medicines list^9^, the fundamental changes to treatment of mild asthma first included in the ground-breaking 2019 GINA report^10^ may provide a feasible solution to reduce the risk of severe exacerbations with very low dose treatment.

In this review we discuss four key concepts for asthma management in primary care: diagnosis, long-term treatment, assessment of control, and management of severe asthma. We provide the background to the latest (May 2022) update of the GINA strategy report^11^, with a focus on changes (Table 1) and selected recommendations that are particularly pertinent to primary care practitioners, and their rationale. The full strategy documents, podcasts, educational materials, and summary booklets are available on the GINA website (https://ginasthma.org).

**Table 1:** Summary of changes in the 2022 GINA Strategy Report of particular relevance to primary care.

Topic or section	Changes
Diagnosis of asthma	Diagnostic testing is different depending on whether the patient is already on controller treatment or is treatment-naive or taking SABA alone (see Tables 2 and 3). Detail has been included about diagnosis and management of asthma in low-resource settings
Assessment of symptom control	When assessing symptom control, record how often the patient is using their reliever inhaler (ICS–formoterol or SABA). For patients prescribed a SABA reliever, use of SABA more than two days a week should prompt review of their adherence and inhaler technique with their maintenance controller treatment. This criterion does not apply to patients using an ICS–formoterol reliever, as it is providing additional controller treatment along with the symptom relief. Dispensing of three or more SABA canisters a year (more than average 1.5 puffs/day) is associated with increased risk of severe exacerbations, and may be associated with increased risk of asthma death
Definition of mild asthma	GINA suggests that the term ‘mild asthma’ should generally be avoided in clinical practice where possible, because patients often assume that it means they do not need any controller treatment. However, if the term is used, explain to the patient that patients with apparently mild asthma can still have severe attacks, and that using ICS-containing treatment, especially with ICS–formoterol reliever, will markedly reduce this risk
GINA treatment figure for adults and adolescents	The rationale for showing two treatment tracks has been reinforced: Track 1, with as-needed ICS–formoterol as reliever across treatment steps, is preferred based on evidence for lower risk of exacerbations and similar or better symptom control compared with using SABA as reliever
Treatment figure for children 6–11 years	The figure has been updated to explain the “other controller options” and new Step 5 options for this age group
Adding LAMA to ICS-LABA for adults and adolescents (Step 5)	Patients with exacerbations despite ICS-LABA should receive at least medium dose ICS-LABA before considering add-on LAMA
Difficult-to-treat and severe asthma in adults and adolescents	The GINA Guide and decision tree for assessment and management of difficult-to-treat and severe asthma in adults and adolescents has been revised and enlarged. Additional investigations have been suggested for patients with difficult-to-treat asthma and blood eosinophils ≥300/μL, including investigating for non-asthma causes such as Strongyloides, which is often asymptomatic. New biologic treatment options have been approved for severe asthma and are available in many countries, so referral to a specialist is recommended if asthma is poorly controlled despite Step 4 treatment
Maintenance oral corticosteroids—consider only as last resort	Because of the risk of serious long-term adverse effects, maintenance OCS should be considered only as a last resort in any age group
Written asthma action plans (handwritten, printed, digital, or pictorial)	Give patients documented instructions about how to change their medications when their asthma worsens, and when to seek medical advice. Verbal instructions are often forgotten
Management of wheezing episodes in pre-school children	In children ≤5 years with intermittent viral wheezing and no or few interval respiratory symptoms, consideration of intermittent short-course ICS has been added to the treatment figure. It should be considered only if the physician is confident that it will be used appropriately, because of the risk of side effects
Management of acute asthma in healthcare settings	After an Emergency Department visit or hospitalization, make sure patients are returned to as-needed (rather than regular) reliever use. For patients using ICS–formoterol as their reliever, make sure that they switch back to this after any acute healthcare presentation

Modified with permission from ref. ^11^.

*ICS* inhaled corticosteroid, *LABA* long-acting beta_2_ agonist, *LAMA* long-acting muscarinic antagonist, *SABA* short-acting beta_2_ agonist, *OCS* oral corticosteroid.

## Diagnosis of asthma

### It is critical to confirm the diagnosis of asthma

Primary care clinicians are consulted by patients with many hundreds of different medical conditions in any year. Every day, they are faced with the challenge of quickly arriving at an accurate diagnosis in limited time, and often with limited access to specialized investigations.

In order to ensure diagnosis of asthma is considered as early as possible, clinicians should maintain a high index of suspicion when patients present with respiratory symptoms^12^.

Over- and under-diagnosis of asthma are common and are usually due to the lack of objective lung function testing which can demonstrate variable expiratory airflow limitation that will support the diagnosis of asthma and help to exclude other causes^13,14^. For continuity of care, it is important to ensure that the diagnosis is recorded in each patient’s medical record, detailing the basis for the diagnosis, including objective measurements of variable airflow obstruction and airway inflammation, if available. These details are often lacking in the medical records of children^15^ and adults treated for asthma^16,17^.

Medical records should also contain details of treatment prescribed, education given to help patients understand the chronic nature of their disease, and provision of a personal written action plan to enable them to change their treatment and seek assistance when needed.

#### Diagnosing asthma in adults, adolescents and children aged 6–11 years

##### Confirm the diagnosis of asthma before starting controller treatment, if possible

**Fig. 1:**
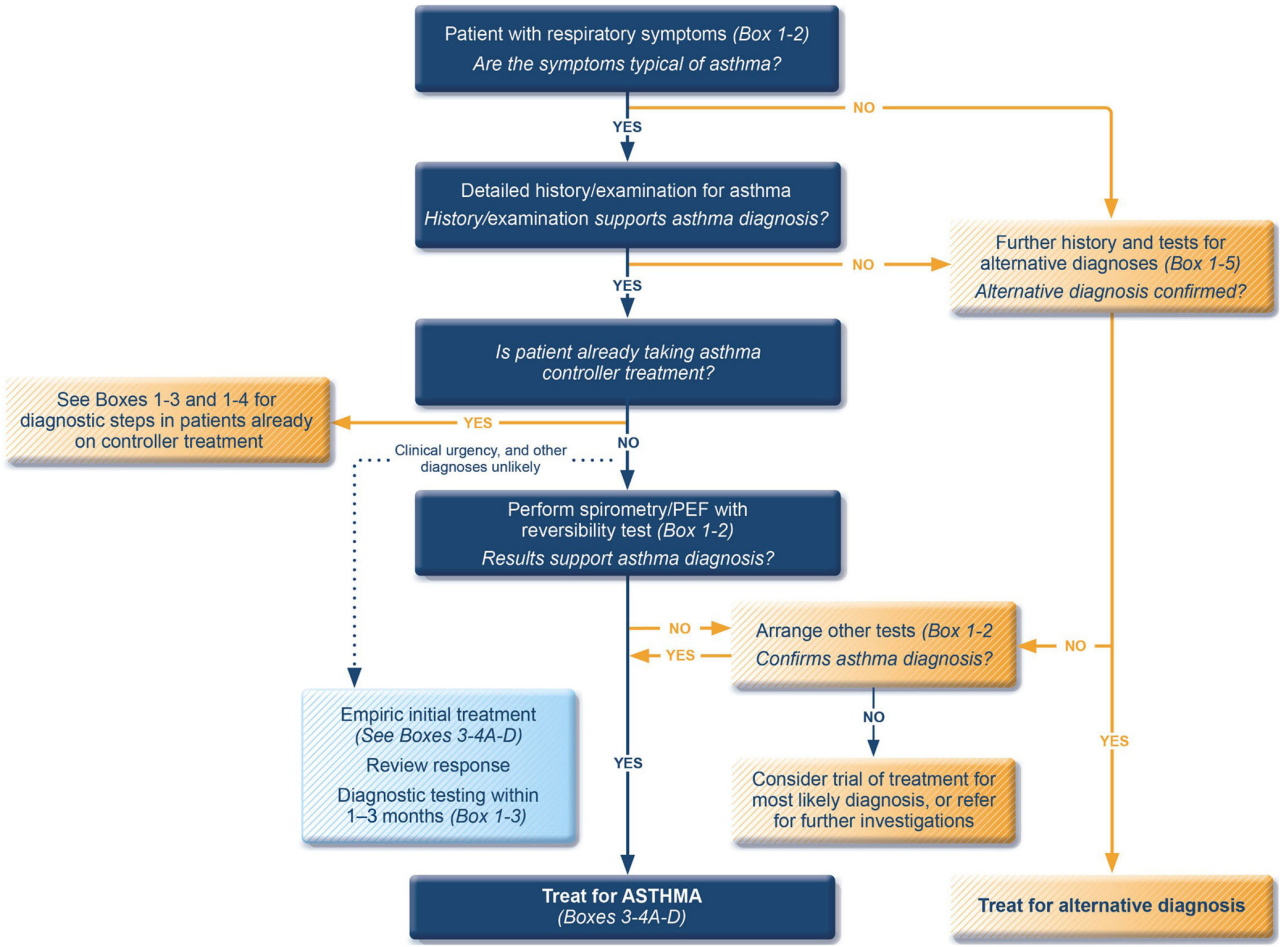
The GINA diagnostic flowchart 2022. PEF peak expiratory flow. Source: Box 1–1 in GINA report 2022. Box numbers within the figure refer to the GINA 2022 report. Reproduced with permission from ref. ^11^.

There is no single test for confirming the diagnosis of asthma. First, a clinical diagnosis starts with a history of respiratory symptoms (such as cough, wheeze, difficulty breathing and/or shortness of breath) that typically vary over time and intensity (Table 2 and Fig. 1). Symptoms of asthma are often worse at night and in the early morning, and may be triggered by factors such as viral infections, allergen exposure, exercise, strong smells, cigarette smoke, exhaust fumes and laughter. When taking a history, it may be helpful to show patients or carers a video depicting typical symptoms, such as the one developed by Wellington School of Medical and Health Sciences, University of Otago, New Zealand, available from the Global Asthma Network website (http://globalasthmanetwork.org/surveillance/manual/Asthma_AVQ3.1.mp4). Physical examination may be entirely normal.

**Table 2:** Diagnostic criteria for asthma in adults, adolescents, and children 6–11 years.

**1. HISTORY OF VARIABLE RESPIRATORY SYMPTOMS**	
*Feature*	*Symptoms or features that support the diagnosis of asthma*
**Wheeze, shortness of breath, chest tightness and cough** (Descriptors may vary between cultures and by age)	• More than one type of respiratory symptom (in adults, isolated cough is seldom due to asthma) • Symptoms occur variably over time and vary in intensity • Symptoms are often worse at night or on waking • Symptoms are often triggered by exercise, laughter, allergens, cold air • Symptoms often appear or worsen with viral infections
**2. CONFIRMED VARIABLE EXPIRATORY AIRFLOW LIMITATION**	
*Feature*	*Considerations, definitions, criteria*
2.1 Documented* expiratory airflow limitation	At a time when FEV_1_ is reduced, confirm that FEV_1_/FVC is reduced compared with the lower limit of normal (it is usually >0.75–0.80 in adults, >0.90 in children)
**AND**	
2.2 Documented* excessive variability in lung function* (one or more of the following):	The greater the variations, or the more occasions excess variation is seen, the more confident the diagnosis. If initially negative, tests can be repeated during symptoms or in the early morning.
• Positive bronchodilator (BD) responsiveness (reversibility) test	*Adults*: increase in FEV_1_ of >12% and >200 mL (greater confidence if increase is >15% and >400 mL). *Children*: increase in FEV_1_ by >12% predicted Measure change 10–15 min after 200–400 mcg salbutamol (albuterol) or equivalent, compared with pre-BD readings. Positive test more likely if BD withheld before test: SABA ≥ 4 h, twice-daily LABA 24 h, once-daily LABA 36 h
• Excessive variability in twice-daily PEF over 2 weeks	*Adults*: average daily diurnal PEF variability >10%^a^ *Children*: average daily diurnal PEF variability >13%^a^
• Significant increase in lung function after 4 weeks of anti-inflammatory treatment	*Adults*: increase in FEV_1_ by >12% and >200 mL (or PEF^2^ by >20%) from baseline after 4 weeks of treatment, outside respiratory infections
• Positive exercise challenge test	*Adults*: fall in FEV_1_ of >10% and >200 mL from baseline *Children*: fall in FEV_1_ of >12% predicted, or PEF >15%
• Positive bronchial challenge test (usually only for adults)	Fall in FEV_1_ from baseline of ≥20% with standard doses of methacholine, or ≥15% with standardized hyperventilation, hypertonic saline or mannitol challenge
• Excessive variation in lung function between visits (good specificity but poor sensitivity)	*Adults*: variation in FEV_1_ of >12% and >200 mL between visits, outside of respiratory infections *Children*: variation in FEV_1_ of >12% in FEV_1_ or >15% in PEF^b^ between visits (may include respiratory infections)

Source: Box 1–2 in GINA 2022. Reproduced with permission from ref. ^11^.

*BD* bronchodilator (SABA or rapid-acting LABA), *FEV*_*1*_ forced expiratory volume in 1 s, *ICS* inhaled corticosteroid, *LABA* long-acting beta_2_ agonist, *PEF* peak expiratory flow (highest of three readings), *SABA* short-acting beta_2_ agonist.

^a^Daily diurnal PEF variability is calculated from twice daily PEF as (day’s highest minus day’s lowest) divided by (mean of day’s highest and lowest), averaged over 1 week.

^b^Use the same PEF meter each time, as PEF may vary by up to 20% between different meters.

Variable expiratory airflow limitation is the other cardinal feature of untreated asthma. In a patient with a history suggestive of asthma, the diagnosis of asthma is supported by an increase in forced expiratory volume in 1 s (FEV_1_) recorded by spirometry 15 min after administration of bronchodilator: in adults/adolescents, by an increase of more than 200 mL and 12% from the pre-bronchodilator (baseline) FEV_1_; in children, by an increase from baseline of more than 12% of the predicted FEV_1_ value.

Since asthma is a variable condition, bronchodilator reversibility (also called responsiveness) may or may not be present at the time of initial lung function testing. If it is not documented on spirometry at an initial attempt, the test should be repeated at one or more later visits, preferably when the patient is symptomatic and bronchodilator medicines have been withheld. Otherwise, an alternative test may be conducted (as below and in Table 2).

Spirometry is not always accessible in primary care. An alternative method is to instruct the patient to record peak expiratory flow (PEF) each morning and evening over a 2-week period in a diary or using an electronic peak flow meter. PEF should be measured three times on each occasion, and only the highest reading used. Diurnal PEF variability is calculated as each day’s highest minus the day’s lowest reading, divided by the mean of the day’s highest and lowest, then these results are averaged over one week. Excessive diurnal PEF variability is defined as a mean variability of >10% in PEFs in adults or >13% variability in children. When measuring PEF, the same meter should be used for all readings, as variation between different PEF meters may be as large as 20%.

In people with suspected asthma who have normal expiratory airflow and no significant reversibility, a bronchoprovocation test (e.g., methacholine or mannitol) can reveal airway hyperresponsiveness, supporting a diagnosis of asthma. Bronchodilators must be withheld before challenge testing.

Variable expiratory airflow limitation should preferably be demonstrated before initiating asthma controller treatment, except in situations of clinical urgency, as it becomes harder to confirm the diagnosis once controller treatment has been started (Table 3). However, the diagnosis of asthma can also be confirmed if there is a clinically significant improvement in FEV_1_ (by >12% and >200 mL) or in PEF by >20% after 4 weeks of inhaled corticosteroid (ICS) treatment.

**Table 3:** Steps for confirming the diagnosis of asthma in a patient already taking controller treatment.

Current status	Steps to confirm the diagnosis of asthma
Variable respiratory symptoms and variable airflow limitation	Diagnosis of asthma is confirmed. Assess the level of asthma control (Box 2–2) and review controller treatment (Box 3–5).
Variable respiratory symptoms but no variable airflow limitation	Consider repeating spirometry after withholding BD (4 h for SABA, 24 h for twice-daily ICS-LABA, 36 h for once-daily ICS-LABA) or during symptoms. Check between-visit variability of FEV_1_, and bronchodilator responsiveness. If still normal, consider other diagnoses (Box 1–5). *If FEV*_*1*_ *is* >*70% predicted*: consider stepping down controller treatment (see Box 1–5) and reassess in 2–4 weeks, then consider bronchial provocation test or repeating BD responsiveness. *If FEV*_*1*_ *is* <*70% predicted*: consider stepping up controller treatment for 3 months (Box 3–5), then reassess symptoms and lung function. If no response, resume previous treatment and refer patient for diagnosis and investigation.
Few respiratory symptoms, normal lung function, and no variable airflow limitation	Consider repeating BD responsiveness test again after withholding BD as above or during symptoms. If normal, consider alternative diagnoses (Box 1–5). Consider stepping down controller treatment (see Box 1–5): • *If symptoms emerge and lung function falls*: asthma is confirmed. Step up controller treatment to previous lowest effective dose. • *If no change in symptoms or lung function at lowest controller step*: consider ceasing controller, and monitor patient closely for at least 12 months (Box 3–7).
Persistent shortness of breath and persistent airflow limitation	Consider stepping up controller treatment for 3 months (Box 3–5), then reassess symptoms and lung function. If no response, resume previous treatment and refer patient for diagnosis and investigation. Consider asthma–COPD overlap (Chapter 5).

“Variable airflow limitation” refers to expiratory airflow. GINA recommendations for confirming the diagnosis in those already started on controller treatment. Source: Box 1–3 in GINA 2022. Box and chapter numbers refer to the GINA 2022 report. Reproduced with permission from ref. ^11^.

*BD* bronchodilator, *COPD* chronic obstructive pulmonary disease, *FEV*_*1*_ forced expiratory volume in 1 s, *ICS* inhaled corticosteroid, *LABA* long-acting beta_2_ agonist, *SABA* short-acting beta_2_ agonist.

A history or family history of allergic rhinitis or atopic dermatitis, or the presence of atopy (demonstrated by either a positive skin prick test or specific IgE to one or more aeroallergens) increases the chance that a patient with respiratory symptoms has allergic asthma, but these features are not specific for asthma, and asthma may be non-allergic.

Evidence of Type 2 inflammation (for example, fractional exhaled nitric oxide [FeNO] >25 ppb or blood eosinophils >300/μL) is found in some types of asthma, but also in several non-asthma conditions such as allergic rhinitis and eosinophilic bronchitis. Therefore, the presence or absence of these biomarkers cannot confirm or exclude a diagnosis of asthma, particularly if measured after starting ICS treatment. However, in patients with severe asthma, FeNO and blood eosinophils are useful to select and guide treatment.

**Fig. 2:**
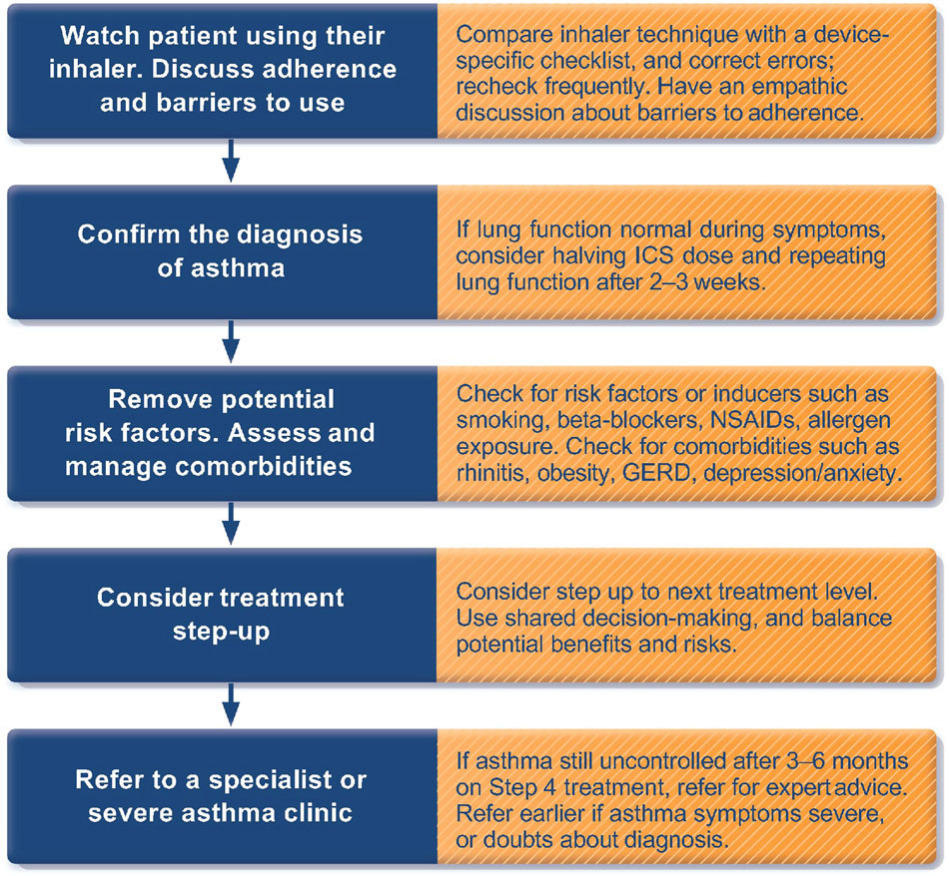
Investigating poor symptom control and/or exacerbations despite treatment. ICS inhaled corticosteroid, NSAID nonsteroidal anti-inflammatory drug, GERD gastro-esophageal reflux disease. Reproduced with permission from ref. ^11^.

If symptoms persist or are more typical of an alternative diagnosis, or if the patient experiences no benefit after commencement of controller therapy, the diagnosis should be reviewed, and alternative causes of the symptoms should be considered (Fig. 2).

##### Consider occupational asthma in patients presenting with adult-onset asthma

Occupational asthma should be considered in anyone newly presenting in adulthood with symptoms suggestive of asthma, particularly if there is improvement when away from work. If occupational asthma is suspected, early referral to a specialist (if available) is important, to assist with assessment of the person’s work environment and confirm the diagnosis. Exposure to the sensitizing agent should cease if at all possible, because ongoing exposure to even low levels can lead to severe problems. The specialist may be able to assist with negotiation with employers to reduce/cease exposure and, where relevant, with recommendations for compensation in accordance with applicable local employment laws. Patients with adult-onset asthma should also be asked about exposure to sensitizers or irritants in non-work locations, e.g., use of cleaning agents at home, or hobbies such as woodworking.

##### Persistent airflow obstruction may develop over time—so it is important to differentiate asthma from chronic obstructive pulmonary disease (COPD)

The history and pattern of symptoms and past records can help to distinguish asthma with persistent airflow limitation from COPD. Asthma and COPD may co-exist in the same patient, particularly in smokers and the elderly.

**Fig. 3:**
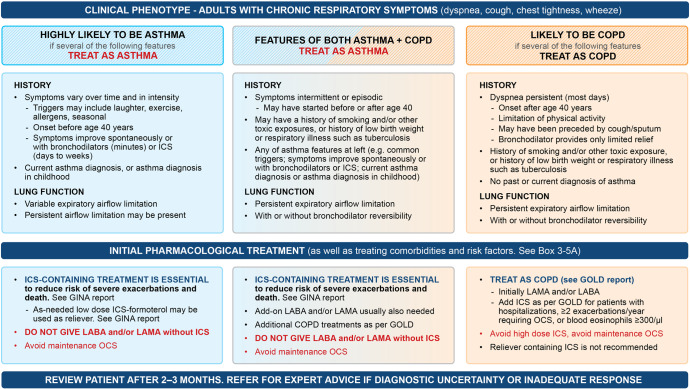
Approach to initial treatment in patients with asthma and/or COPD. GOLD Global Initiative for Obstructive Lung Disease, ICS inhaled corticosteroid, LABA long-acting β_2_ agonist; LAMA long-acting muscarinic antagonist. A summary of differentiating and diagnostic features in people with Asthma, COPD and Asthma + COPD. Source: Box 5–2 in GINA 2022. Reproduced with permission from ref. ^11^.

It is important to recognize features of asthma in these patients because anti-inflammatory treatment with ICS is essential in asthma (whether or not there are also features of COPD such as persistent airflow limitation) to prevent severe flare-ups (severe exacerbations) and reduce the risk of asthma-related death. Figure 3 summarizes features that are useful in distinguishing asthma from COPD.

#### Diagnosing asthma in children aged 5 years and under

It can be challenging to make the diagnosis of asthma in some children aged ≤5 years. Recurrent wheezing is very common in this age group, including in children without asthma, typically with viral upper respiratory tract infections. Routine assessment of airflow limitation or bronchodilator responsiveness in this age group is difficult and is not practical in primary care.

##### Asthma diagnosis in children aged ≤5 years can be based on symptom patterns, the presence of risk factors, therapeutic response to controller treatment, and exclusion of alternative diagnoses

A diagnosis of asthma in young children with a history of wheezing is more likely if they have wheezing or coughing that occurs with exercise, laughing or crying, or in the absence of an apparent respiratory infection, a history of other allergic disease (eczema, food allergy, or allergic rhinitis), atopy or asthma in first-degree relatives, clinical improvement during 2–3 months of controller treatment, and worsening after cessation.

The following questions can be used to elicit features suggestive of asthma in young children and features that help support the diagnosis:

- Does your child have wheezing? Wheezing is a high-pitched noise that comes from the chest and not the throat. Use of a video questionnaire, or asking a parent to record an episode on a smartphone if available can help to confirm the presence of wheeze and differentiate from upper airway abnormalities.
- Does your child wake up at night because of coughing, wheezing, or difficult breathing, heavy breathing, or breathlessness?
- Does your child have to stop running, or play less hard, because of coughing, wheezing or difficult breathing, heavy breathing, or shortness of breath?
- Does your child cough, wheeze or get difficult breathing, heavy breathing, or shortness of breath when laughing, crying, playing with animals, or when exposed to strong smells or smoke?
- Has your child ever had eczema, or been diagnosed with allergy to foods?
- Has anyone in your close family had asthma, hay fever, food allergy, eczema, or any other disease with breathing problems?

In preschool children with wheeze, phenotypes have been proposed based on short-term symptom patterns^18^ or on symptom pattern trends over time^19–21^, but these have not proved to be clinically useful or accurate in predicting asthma in later childhood.

## Long-term treatment of asthma

### All patients diagnosed with asthma should be treated with ICS-containing medication

GINA recommends that all adults, adolescents and children over 5 years with a diagnosis of asthma should be treated with regular or (for mild asthma) as-needed ICS-containing treatment to control symptoms and prevent flare-ups (also called exacerbations or “attacks”), and that they should be reviewed within three months after initiating and/or changing treatment. In children ≤5 years, ICS treatment is recommended if asthma is likely and the child has uncontrolled symptoms and/or ≥3 wheezing episodes/year; a trial of ICS is also recommended if the diagnosis is uncertain and symptoms occur more than every 6–8 weeks.

### GINA recommends against treating asthma with SABA alone, without ICS

**Fig. 4:**
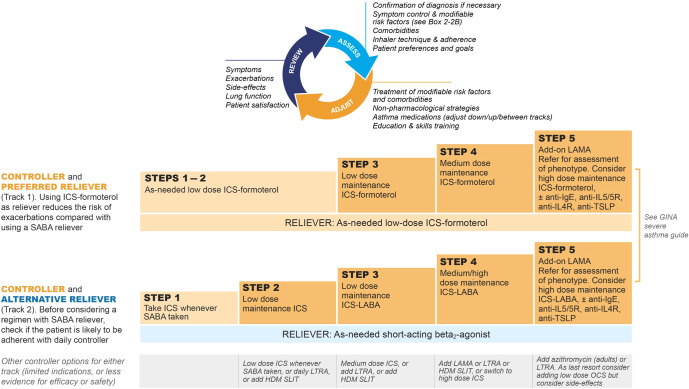
Two-track options for personalized management of asthma for adults and adolescents, to control symptoms and minimize future risk. HDM house dust mite, ICS inhaled corticosteroid, LABA long-acting beta_2_ agonist, LAMA long-acting muscarinic antagonist, LTRA leukotriene receptor antagonist, OCS oral corticosteroids, SABA short-acting beta_2_ agonist, SLIT sublingual immunotherapy. Box number refers to the GINA 2022 report. Before starting, stepping up or down or switching between tracks, patients should be assessed using the “assess, adjust, review” cycle shown at the top of the figure. Refer to the GINA report for more information about Step 5 options, including biologic therapies for patients with severe asthma. Source: Box 3–5A in GINA report 2022. Reproduced with permission from ref. ^11^.

**Fig. 5:**
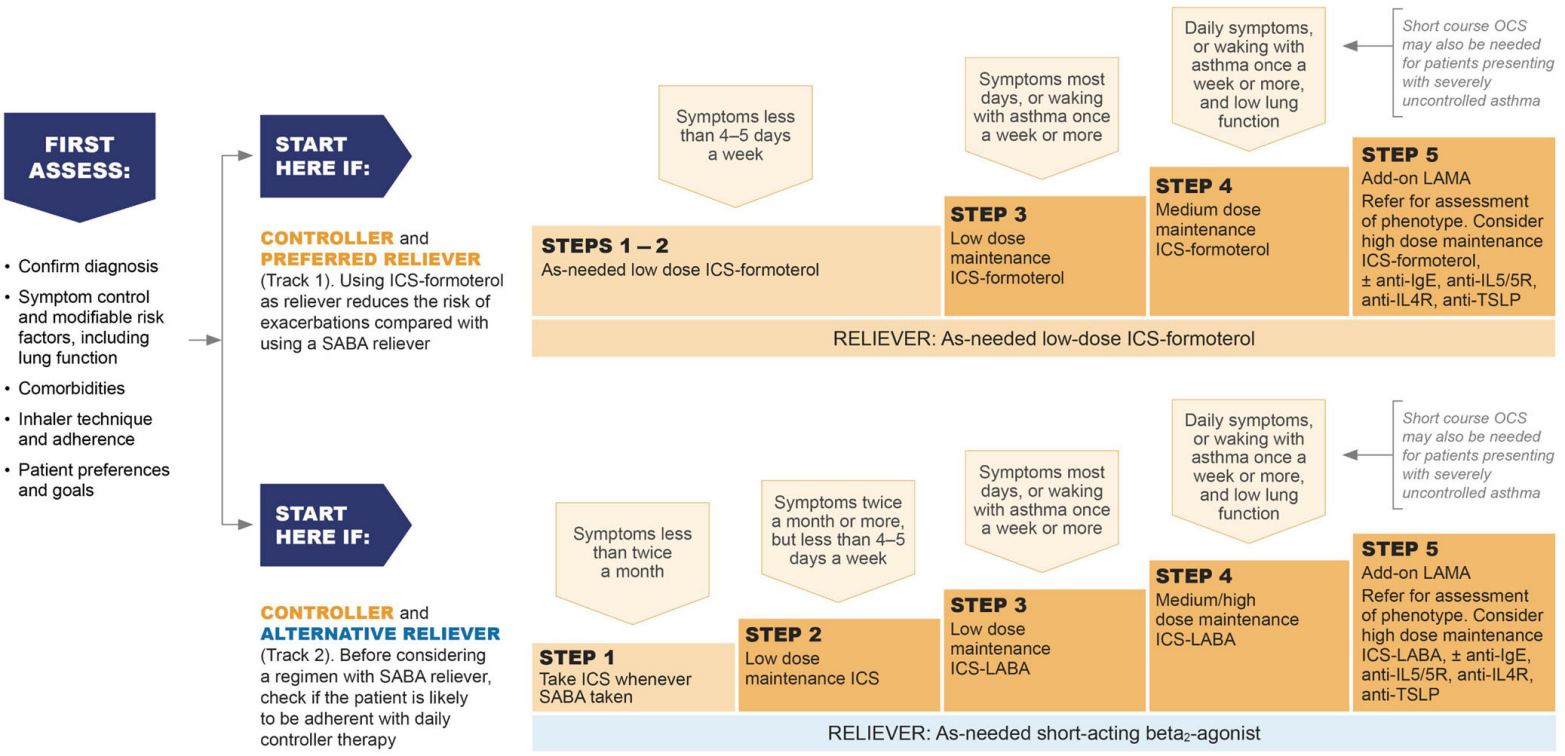
Initial medications for adults and adolescents diagnosed with asthma. ICS inhaled corticosteroid, LABA long-acting beta_2_ agonist, LAMA long-acting muscarinic antagonist, MART maintenance and reliever therapy with ICS–formoterol, OCS oral corticosteroids, SABA short-acting beta_2_ agonist. Initial medications for adults and adolescents diagnosed with asthma, with guidance on initial levels of medication for each treatment track based on symptoms and lung function where appropriate. Refer to the GINA report for other treatment components, including treatment of modifiable risk factors and comorbidities, non-pharmacologic strategies, and education and skills training. Source: Box 3.4Bi in GINA report 2022. Reproduced with permission from ref. ^11^.

**Fig. 6:**
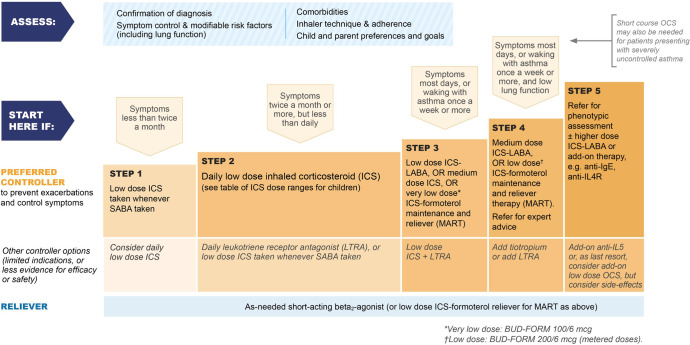
Initial medications for children aged 6–11 years diagnosed with asthma. BUD-FORM budesonide–formoterol, ICS inhaled corticosteroid, LABA long-acting beta_2_ agonist, LTRA leukotriene receptor antagonist, MART maintenance and reliever therapy with ICS–formoterol, OCS oral corticosteroids, SABA short-acting beta_2_ agonist. Initial treatment for children aged 6–11 years diagnosed with asthma, with guidance on initial levels of medication for each treatment track based on symptoms and lung function where appropriate. Source: Box 3–4Di in GINA report 2022. Refer to the GINA report for other treatment components, including treatment of modifiable risk factors and comorbidities, non-pharmacologic strategies, and education and skills training. Reproduced with permission from ref. ^11^.

GINA no longer recommends treatment of asthma with SABA alone (without ICS) in adults, adolescents and children >5 years (Figs. 4–6) because of the risk of severe asthma flare-ups (severe exacerbations) requiring emergency department presentation or hospitalization, and asthma-related death. These risks are markedly reduced by ICS-containing therapy^22,23^. Treating with ICS also substantially reduces the need for courses of oral corticosteroids, thereby reducing the cumulative risk of long-term adverse effects such as osteoporosis and cataract from even occasional courses of oral corticosteroids^24^. A further reason to avoid treating asthma with SABA alone is because their quick symptom relief may instill a false sense of security in patients, who may incorrectly assume that these medicines alone are a sufficient treatment for asthma. In addition, regular use of SABA (e.g., 2–4 times daily for as little as 1–2 weeks) increases airway hyperresponsiveness and airway inflammation^25,26^, and overuse of SABA (indicated by dispensing of ≥3 200-dose canisters in a year, or daily use), is associated with an increased risk of severe exacerbations and death, even in patients also taking ICS^27–29^.

### Compared with as-needed SABA, as-needed low-dose ICS–formoterol for symptom relief reduces the risk of severe asthma flare-ups (severe exacerbations) across all levels of asthma severity—either as-needed only in mild asthma or in addition to maintenance ICS–formoterol

GINA’s current recommendations for the pharmacotherapy of asthma in adults and adolescents are shown in two ‘tracks’ (Figs. 4–5). There is strong evidence favoring the Track 1 option, in which low-dose ICS–formoterol is the reliever across all treatment steps, compared with Track 2, in which SABA is the reliever^23,30–39^.

This recommendation is based on multiple studies demonstrating that combination low-dose ICS and formoterol, taken as-needed for relief of asthma symptoms (either as-needed only in mild asthma, or in addition to maintenance ICS–formoterol), is a more effective and safer reliever than as-needed SABA.

In patients with mild asthma, as-needed ICS–formoterol reduces the risk of severe flare-ups by 60–64% compared with as-needed SABA^36,37^. Compared with low-dose maintenance ICS plus as-needed SABA, the risk of severe exacerbations is similar^35–38^. In a Cochrane systematic review and meta-analysis (*n* = 9565), patients with mild asthma treated with as-needed ICS–formoterol had a 55% reduction in severe exacerbations and 65% lower emergency department visits or hospitalizations compared with SABA alone. In addition, those treated with as-needed ICS–formoterol had 37% lower risk of emergency department visits or hospitalizations than with daily ICS plus as-needed SABA^23^. In some of these studies, there were small differences in lung function (FEV_1_) and symptom control assessed by Asthma Control Questionnaire (ACQ-5) score that favored daily maintenance ICS over as-needed-only low-dose ICS–formoterol. These differences were not clinically important, and may reflect that adherence with maintenance ICS was much higher than is usually achievable in clinical practice. The average daily dose of ICS was much lower with as-needed ICS–formoterol compared with daily ICS plus as-needed SABA.

Further, in patients with moderate-to-severe asthma (Steps 3 and 4, Figs. 4–5), use of ICS–formoterol as both maintenance and reliever therapy (MART) in Track 1 reduces the risk of severe flare-ups (severe exacerbations), compared with taking the same or higher dose of ICS or a combination of ICS and a long-acting beta_2_ agonist (LABA) plus SABA reliever^30,31^. In Steps 3 and 4, symptom control and lung function with MART are the same or better compared with use of a SABA reliever.

Although Track 1 is preferred because of the significant reduction in severe exacerbations, Track 2 (with SABA as reliever) is an alternative option if ICS–formoterol is not available or if patients have no risk factors for exacerbations and have good adherence with regular controller therapy. However, before prescribing Track 2 therapy with a SABA reliever, the clinician should assess whether the patient is likely to continue to be adherent with daily controller treatment, as otherwise they will be taking SABA alone, with an increased risk of severe exacerbations.

At the time of publishing, over 45 countries have licensed ICS–formoterol for as-needed use in mild asthma and over 120 countries have licensed prescription of MART in moderate-to-severe asthma (personal communications). Detailed practical advice on the implementation of MART in clinical practice has recently been published^40,41^, including downloadable resources (ICS–formoterol dosing and SMART action plan).

### GINA asthma treatment is not “one size fits all”

Because asthma is a chronic condition prone to flare-ups, GINA emphasizes that patients need regular review, assessment and adjustment. This involves assessment of asthma control, individual risk factors and comorbidities, with review and optimization of treatment, including careful attention to adherence and inhaler technique, and provision of individualized self-management education including a written/pictorial action plan.

**Fig. 7:**
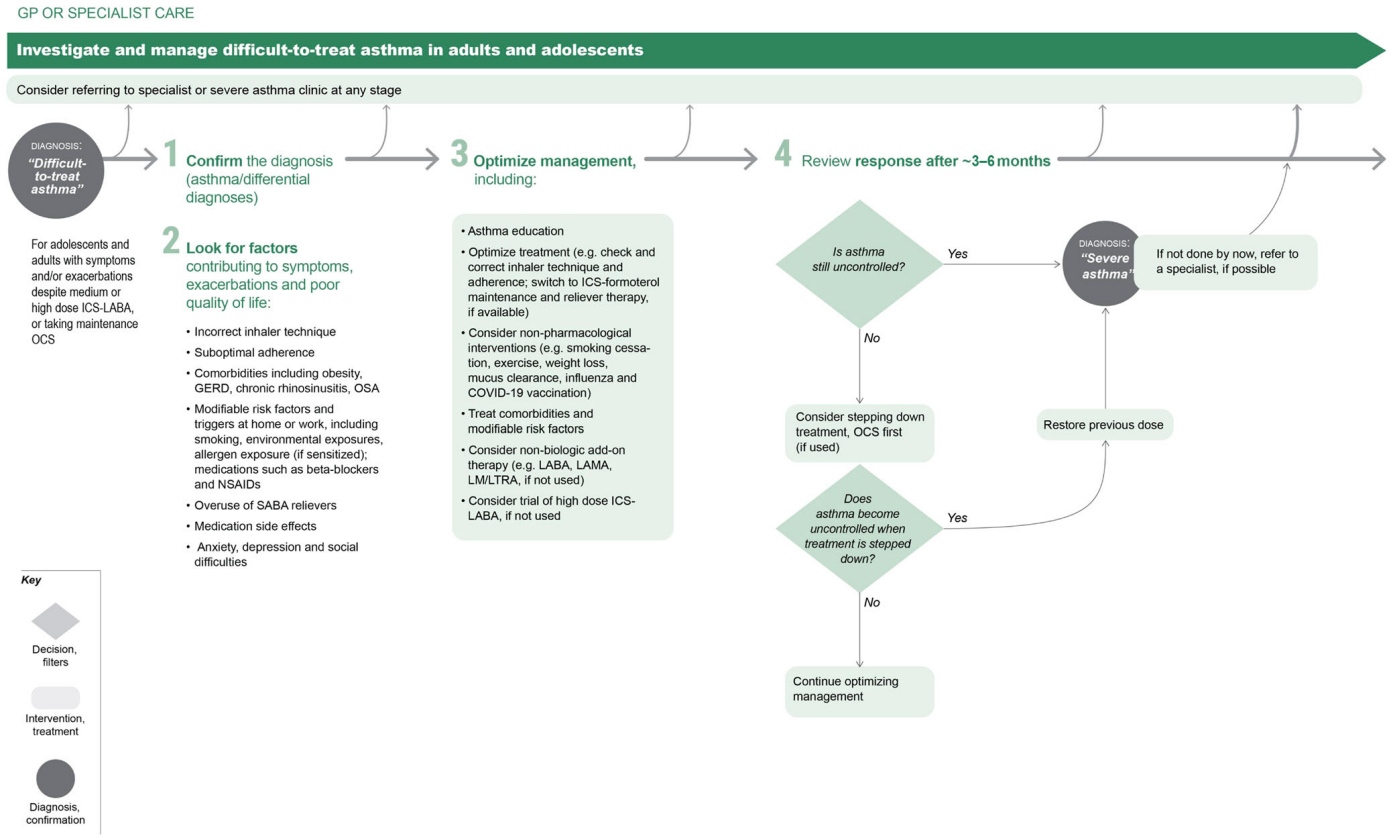
Investigating and managing difficult-to-treat asthma in adult and adolescent patients. ICS inhaled corticosteroid, LABA long-acting beta_2_ agonist, LAMA long-acting muscarinic antagonist, LTRA leukotriene receptor antagonists, SABA short-acting beta_2_ agoinst, OCS oral corticosteroid. The GINA strategy includes a decision tree about the management of difficult-to-treat and severe asthma spanning primary through tertiary care. The section of the flow diagram applicable to generalists in primary and secondary care is shown here. Source: Box 3–16A in GINA report 2022. Reproduced with permission from ref. ^11^.

Management of co-morbid conditions that may worsen asthma control, increase the risk of severe flare-ups and/or complicate treatment should be optimized. These comorbidities include obesity, chronic rhinosinusitis, obstructive sleep apnea, gastro-esophageal reflux disease, and mental health problems (Fig. 7).

Treatment should be reviewed after any flare-ups or changes in treatment (Fig. 2). The components of these assessments are summarized in the personalized asthma management cycle (Assess, Adjust, Review) shown at the top of Fig. 4, which guides clinicians in personalized asthma review and adjustment of treatment. This approach emphasizes the principle that asthma treatment is not ‘one size fits all’.

Management of each patient’s individual risk factors and comorbidities may include both pharmacologic and non-pharmacologic strategies. Non-pharmacologic strategies may include smoking cessation advice, breathing exercises, weight reduction, avoiding air pollution and allergens, appropriate immunizations as well as strategies for dealing with emotional stress. In addition, it is essential to ensure patients can use their prescribed inhaler correctly with reinforcement of approved local videos (e.g., https://www.nationalasthma.org.au/living-with-asthma/how-to-videos). In patients with severe asthma, assessment of inflammatory biomarkers (blood eosinophils and/or FeNO) is important for guiding selection and adjustment of asthma treatment.

A written or pictorial action plan on the management of asthma exacerbations should be provided to every patient. The action plan should be appropriate for the patient’s level of literacy and health literacy, and their treatment regimen. Examples of action plans, including for patients using ICS–formoterol reliever as in GINA Track 1, are available at https://www.nationalasthma.org.au/health-professionals/asthma-action-plans/asthma-action-plan-library. The risk of adverse effects of medications can be reduced by optimizing inhaler technique and adherence, stepping down ICS dose when asthma has been well-controlled for 2–3 months, by referring patients for specialist review (if available) if asthma is not well controlled despite medium or high dose ICS-LABA, and by identifying patients with SABA overuse who may be potentially switched to GINA Track 1 with an ICS–formoterol reliever.

Figures 5 and 6 summarize the GINA options for *initial* asthma medications in adults, adolescents and children 6–11 years newly diagnosed with asthma. Once treatment has been initiated, ongoing medication decisions are based on the same personalized cycle, in which treatment is stepped up and down according to the patient’s needs within a track, using the same reliever. Treatment can also be switched between tracks according to patient needs and preferences. Before any step-up (Fig. 2), it is essential to check adherence to treatment, inhaled technique, relevant comorbidities and risk factors, and environmental factors affecting asthma (Supplementary Fig. 1).

## Assessment of asthma control in two domains: symptoms and risk factors

### GINA defines asthma control in two domains: (i) current symptom control and (ii) risk factors for future poor asthma outcomes

People with asthma should be assessed regularly, including after flare-ups. Unfortunately, in many cases, asthma is managed as though it were an acute illness; patients are treated for flare-ups and then sent home without follow-up^42–46^.

Patient-reported tools for assessing asthma symptom control (e.g., Asthma Control Questionnaire, Asthma Control Test, Childhood Asthma Control Test) reflect only the past 1–4 weeks, and therefore provide only a snapshot of recent symptoms, not overall asthma control.

Poor symptom control is associated with an increased risk of asthma flare-ups. However, people with good symptom control or seemingly mild asthma can still be at risk of severe flare-ups (severe exacerbations)^47^, and even death^48^. GINA therefore recommends that asthma control should be assessed in two domains: (i) current symptom control and (ii) risk factors for future poor asthma outcomes, particularly exacerbations (Supplementary Fig. 1).

Supplementary Fig. 1 summarizes how to assess symptom control and provides a list of modifiable risk factors for exacerbations that are independent of the level of symptom control. This means that even if someone has no current or recent symptoms at the time of assessment, they may still be at risk of asthma flare-ups. Treatment of modifiable risk factors may include, for example, correcting inhaler technique, reducing exposure to tobacco smoke, strategies for weight reduction, allergen immunotherapy and/or allergen avoidance in sensitized patients, and arranging mental health support.

Table 4 summarizes specific questions to be addressed when assessing asthma control in children 6–11 years.

**Table 4:** Specific questions to ask when assessing children 6–11 years with asthma.

**Asthma symptom control**	
Day symptoms	Ask: How often does the child have cough, wheeze, dyspnea or heavy breathing (number of times per week or day)? What triggers the symptoms? How are they handled?
Night symptoms	Cough, awakenings, tiredness during the day? (If the only symptom is cough, consider other diagnoses such as rhinitis or gastroesophageal reflux disease).
Reliever use	How often is reliever medication used? (check date on inhaler or last prescription) Distinguish between pre-exercise use (sports) and use for relief of symptoms.
Level of activity	What sports/hobbies/interests does the child have, at school and in their spare time? How does the child’s level of activity compare with their peers or siblings? How many days is the child absent from school? Try to get an accurate picture of the child’s day from the child without interruption from the parent/carer.
**Risk factors for adverse outcomes**	
Exacerbations	Ask: How do viral infections affect the child’s asthma? Do symptoms interfere with school or sports? How long do the symptoms last? How many episodes have occurred since their last medical review? Any urgent doctor/emergency department visits? Is there a written action plan? Risk factors for exacerbations include a history of exacerbations, poor symptom control, poor adherence and poverty, and persistent bronchodilator reversibility even if the child has few symptoms.
Lung function	Check curves and technique. Main focus is on FEV_1_ and FEV_1_/FVC ratio. Plot these values as percent predicted to see trends over time.
Side-effects	Check the child’s height at least yearly, as poorly controlled asthma can affect growth, and growth velocity may be lower in the first 1–2 years of ICS treatment. Ask about frequency and dose of ICS and OCS.
**Treatment factors**	
Inhaler technique	Ask the child to show how they use their inhaler. Compare with a device-specific checklist.
Adherence	Is there any controller medication in the home at present? On how many days does the child use their controller in a week (e.g. 0, 2, 4, 7 days)? Is it easier to remember to use it in the morning or evening? Where is inhaler kept – is it in plain view to reduce forgetting? Check date on inhaler.
Goals/concerns	Does the child or their parent/carer have any concerns about their asthma (e.g. fear of medication, side-effects, interference with activity)? What are the child’s/parent’s/carer’s goals for treatment?
**Comorbidities**	
Allergic rhinitis	Itching, sneezing, nasal obstruction? Can the child breathe through their nose? What medications are being taken for nasal symptoms?
Eczema	Sleep disturbance, topical corticosteroids?
Food allergy	Is the child allergic to any foods? (confirmed food allergy is a risk factor for asthma-related death)
Obesity	Check age-adjusted BMI. Ask about diet and physical activity.
**Other investigations (if needed)**	
2-week diary	If no clear assessment can be made based on the above questions, ask the child or parent/carer to keep a daily diary of asthma symptoms, reliever use and peak expiratory flow (best of three) for 2 weeks (Appendix Chapter 4).
Exercise challenge (laboratory)	Provides information about airway hyperresponsiveness and fitness (Box 1–2). Only undertake a challenge if it is otherwise difficult to assess asthma control.

Source: Box 2–3 in GINA 2022. Box and appendix numbers refer to GINA 2022 report. Reproduced with permission from ref. ^11^.

*FEV*_*1*_ forced expiratory volume over 1 s, *FVC* forced vital capacity, *ICS* inhaled corticosteroid, *OCS* oral corticosteroid.

## Difficult-to-treat and severe asthma

### Refer people with severe asthma to a respiratory specialist, if possible

Difficult-to-treat asthma is defined as asthma that is uncontrolled despite prescribing of medium- or high-dose ICS with a second controller (usually a LABA) or with maintenance oral corticosteroids, or that requires high-dose ICS to maintain good asthma control. For many such patients, their asthma can be well controlled by optimizing care, including identifying and addressing modifiable risk factors listed in Figs. 2 and 7 and Supplementary Fig. 1. Poor adherence and incorrect inhaler technique are particularly common contributors to poor asthma control.

**Fig. 8:**
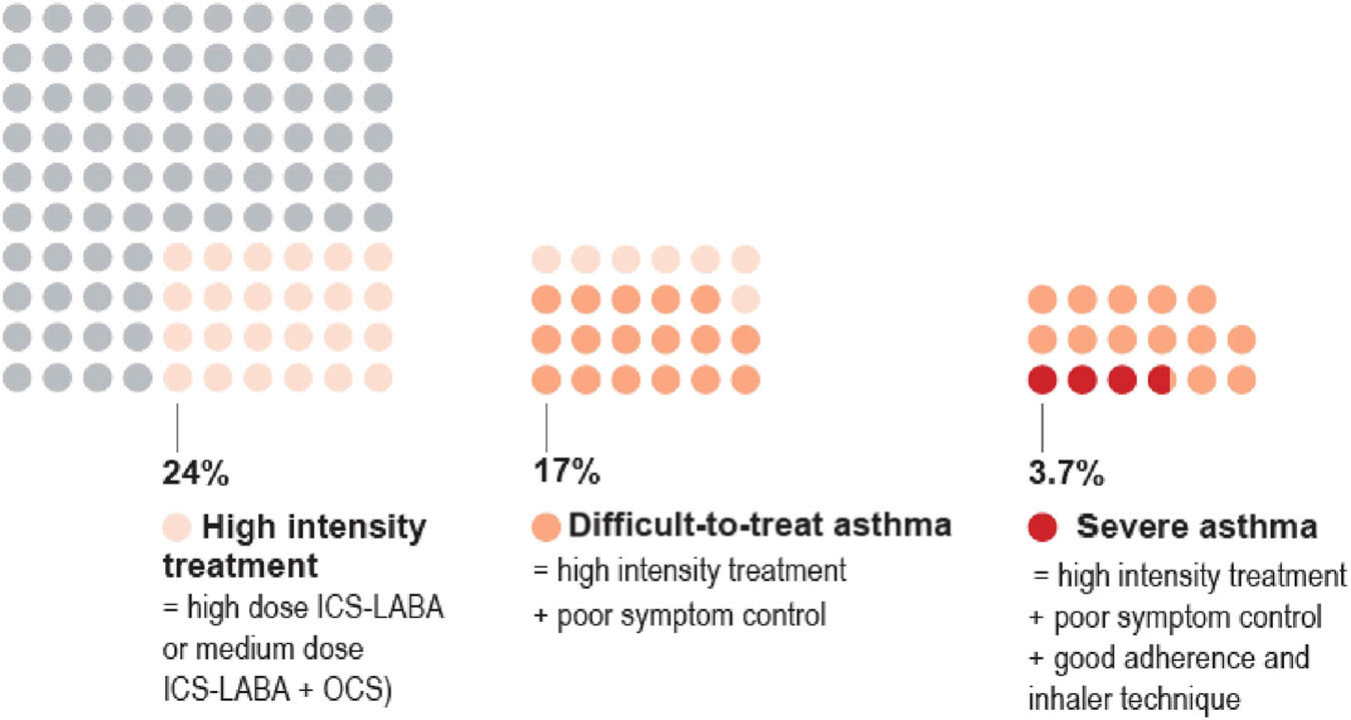
Proportion of adults with difficult-to-treat or severe asthma. Severe asthma is a subset of those with “difficult-to-treat” asthma. Source: Box 3–15 in GINA report 2022, data from Hekking et al.^49^. Reproduced with permission from ref. ^11^.

Severe asthma is a subset of difficult-to-treat asthma (Fig. 8). Severe asthma is defined as asthma that is uncontrolled despite adherence with optimized high-dose ICS-LABA treatment with correct inhaler technique and management of contributory factors such as comorbidities and environment exposures, or that worsens when the dose of ICS-LABA is reduced.

Based on a study in the Netherlands, about 3–4% of people with asthma are estimated to have severe asthma^49^, but many more patients have difficult-to-treat asthma, that could be improved by referral for specialist assessment and treatment^50^. While a small proportion of people with asthma have severe disease, they contribute towards a disproportionately high level of morbidity, mortality and healthcare costs^51,52^.

While most people with asthma can be managed in primary care, it can be challenging to identify those at risk of poor outcomes, and especially those with severe asthma. This difficulty is partly due to the nature of primary care, where large numbers of patients present with many different and often previously undiagnosed medical conditions, there can be severe time pressures, resources may be limited, and at follow-up a patient may see different healthcare professionals with varying levels of expertise or training about asthma. Furthermore, medical records may be poor or incomplete, making it difficult to form a perspective of long-term control and efficacy of treatments, and specifically correctly identifying those that may benefit from specialist referral.

When asthma is poorly controlled despite medium or high dose ICS-LABA, the patient should be reassessed. This involves first ensuring that the diagnosis of asthma has been confirmed and relevant comorbidities and risk factors managed, that ICS have been prescribed, and that asthma treatment has been optimized; that is, that the patient is collecting and using the medication and that they are satisfied with^53^ and are able to use their inhaler correctly^54,55^.

Figure 7 shows other factors and interventions that can also be considered in primary care. If asthma remains uncontrolled, there are several reasons why these people should be referred (if possible) for expert assessment, advice and/or provision of medication, and for guidance on ongoing primary care management. In addition to confirming the diagnosis, specialist asthma services have knowledge of, and access to, newer and specific treatment including the latest range of biologic treatments (monoclonal antibodies for severe asthma). They may also have access to specialist nursing, pharmacists, counseling and psychology expertise and the facilities to provide long-term follow-up and access to consistent support from liaison nurses.

While some primary care clinics may have such expertise and resources, most do not. A number of UK coronial inquests on asthma deaths in children concluded that lack of access to continuity of care contributed to these deaths^42–44^.

The section of the GINA 2022 report on severe asthma diagnosis and management spans the roles of clinicians ranging from primary to tertiary care. Figure 7 summarizes the initial approach to these patients in a primary care setting. The full severe asthma recommendations (including for biologic therapy) as well as a summary booklet are also available on the GINA website.

While most patients’ asthma can be managed in primary care, specialist opinion and treatment is strongly recommended (where available) in some situations:

- when the diagnosis is difficult; specialists will have access to more sophisticated investigations and resources for confirming or excluding a diagnosis of asthma;
- when there is failure to control symptoms despite adequate therapy, good adherence and good inhaler technique;
- when severe asthma is suspected, for characterization of phenotype and for consideration of biologic therapy, depending on availability. For example, primary care physicians should consider referral for patients taking maintenance oral corticosteroids and those who have had two or more courses of oral corticosteroids for acute exacerbations in the previous year, and those who have poorly controlled asthma despite step 4 treatment;
- when symptoms suggest complications or comorbidities such as aspirin-exacerbated respiratory disease, allergic bronchopulmonary aspergillosis;
- when occupational asthma is suspected;
- when a patient has a history of a life-threatening asthma attack, or has confirmed or suspected food allergy as well as asthma.

## Conclusion

In summary, the GINA strategy emphasizes that asthma should be considered in the differential diagnosis of anyone presenting with respiratory symptoms, particularly if recurrent and varying in severity. Where possible, the diagnosis of asthma should be confirmed with lung function testing before initiating controller treatment. Asthma control should be assessed in two domains: current symptom control and risk factors for future asthma flare-ups (exacerbations), which include having had a flare-up in the previous 12 months. Asthma treatment for all patients should include ICS: either regularly or (in mild asthma) as needed whenever symptom reliever is taken.

Optimization of asthma treatment includes education and skills training for inhaler technique and adherence, and provision of a written/pictorial asthma action plan. Failure to successfully optimize care in people with severe or difficult-to-treat asthma should prompt careful reassessment—if available, by a specialist with appropriate facilities for diagnosis and interdisciplinary treatment. Collaboration between primary care doctors and respiratory physicians is a key factor in effective asthma management.

## Supplementary information

Online Supplement

## Data Availability

No datasets were generated or analyzed during the current study.
