# Supplementary material for: Key recommendations for primary care from the 2022 Global Initiative for Asthma (GINA) update
Source: NPJ Prim Care Respir Med. 2023 Feb 8;33:7. doi: 10.1038/s41533-023-00330-1 (PMC9907191; doi:10.1038/s41533-023-00330-1)
Supplement: Supplementary file 1 — Online Supplement [file 41533_2023_330_MOESM1_ESM.pdf]

Online supplement for "Key recommendations for primary care from the 2022 Global Initiative for Asthma (GINA) update"

Mark L Levy*[1], Leonard B Bacharier[2], Eric Bateman[3], Louis-Philippe Boulet[4], Chris Brightling[5], Roland Buhl[6], Guy Brusselle[7], Alvaro A. Cruz[8], Jeffrey M. Drazen M.D.[9], Liesbeth Duijts[10], Louise Fleming[11], Hiromasa Inoue[12], Fanny WS Ko[13], Jerry A. Krishnan, MD, PhD[14], Kevin Mortimer[15], Paulo M Pitrez[16], Aziz Sheikh[17], Arzu Yorgancıoğlu[18], Helen K Reddel[19]

1. Locum general practitioner, London, UK..
2. Professor of Pediatrics, Vanderbilt University Medical Center, Nashville, TN, USA.
3. Department of Medicine, University of Cape Town, Cape Town, South Africa.
4. Québec Heart and Lung Institute, Université Laval, Québec, Canada.
5. Institute for Lung health, Leicester NIHR BRC, University of Leicester, UK.
6. Pulmonary Department, Mainz University Hospital, Mainz, Germany
7. Department of Respiratory Medicine, Ghent University Hospital, Ghent, Belgium and Departments of Epidemiology and Respiratory Medicine, Erasmus Medical Center, University Medical Center Rotterdam, Rotterdam, The Netherlands
8. ProAR Foundation and Federal University of Bahia, Salvador, Bahia, Brazil
9. Senior Physician, Brigham and Women's Hospital and Distinguished Parker B. Francis Professor of Medicine, Harvard Medical School.
10. Department of Pediatrics, divisions of Respiratory Medicine and Allergology, and Neonatology, Erasmus MC, University Medical Center Rotterdam, Rotterdam, Netherlands
11. National Heart and Lung Institute, Imperial College, London.
12. Department of Pulmonary Medicine, Kagoshima University Graduate School of Medical and Dental Sciences, Kagoshima, Japan.
13. Department of Medicine and Therapeutics, The Chinese University of Hong Kong.
14. Breathe Chicago Center, University of Illinois Chicago, Chicago, IL, USA.
15. Liverpool University Hospitals NHS Foundation Trust and University of Cambridge, UK and Department of Paediatrics and Child Health, College of Health Sciences, School of Clinical Medicine, University of KwaZulu-Natal, Durban, South Africa.
16. Hospital Santa Casa de Porto Alegre, Porto Alegre, Brazil.
17. Professor of Primary Care Research & Development and Director, Usher Institute, University of Edinburgh, Edinburgh, UK.
18. Department of Pulmonology, Celal Bayar University, Manisa, Turkey.
19. The Woolcock Institute of Medical Research and The University of Sydney, Sydney, Australia.

Assessment of asthma control

The level of asthma control is the extent to which the features of asthma can be observed in the patient, or have been reduced or removed by treatment.

Asthma control is assessed in two domains: symptom control (Supplementary Figure 1A below) and risk of adverse outcomes (Supplementary Figure 1B).

Poor symptom control is burdensome to patients and increases the risk of exacerbations, but patients with good symptom control can still have severe exacerbations. See GINA 2022 Box 3-8 for strategies to treat modifiable risk factors for exacerbations.

Supplementary Figure 1. GINA assessment of asthma control in adults, adolescents and children 6–11 years

A. Asthma symptom control		Level of asthma symptom control		
**In the past 4 weeks, has the patient had:**		**Well controlled**	**Partly controlled**	**Uncontrolled**
• Daytime asthma symptoms more than twice/week?	Yes☐ No☐	None of these	1–2 of these	3–4 of these
• Any night waking due to asthma?	Yes☐ No☐			
• SABA reliever for symptoms more than twice/week?*	Yes☐ No☐			
• Any activity limitation due to asthma?	Yes☐ No☐			

B. Risk factors for poor asthma outcomes

Assess risk factors at diagnosis and periodically, particularly for patients experiencing exacerbations.

Measure FEV_1 at start of treatment, after 3–6 months of controller treatment to record the patient's personal best lung function, then periodically for ongoing risk assessment.

Having uncontrolled asthma symptoms is an important risk factor for exacerbations.

Additional **potentially modifiable risk factors for flare-ups (exacerbations)**, even in patients with few symptoms† include:

- ***Medications***: high SABA use (≥3x200-dose canisters/year associated with increased risk of exacerbations; increased mortality particularly if ≥1 canister per month); inadequate ICS: not prescribed ICS; poor adherence; incorrect inhaler technique
- ***Other medical conditions***: obesity; chronic rhinosinusitis; GERD; confirmed food allergy; pregnancy
- ***Exposures***: smoking; e-cigarettes; allergen exposure if sensitized; air pollution
- ***Context***: major psychological or socioeconomic problems
- ***Lung function***: low FEV_1, especially <60% predicted; high BD responsiveness
- ***Type 2 inflammatory markers***: higher blood eosinophils; elevated FeNO (in adults with allergic asthma taking ICS)

Other major independent risk factors for flare-ups (exacerbations)

- Ever intubated or in intensive care unit for asthma
- ≥1 severe exacerbation in last 12 months

Having any of these risk factors increases the patient's risk of exacerbations *even if they have few asthma symptoms*

Risk factors for developing persistent airflow limitation

- History: preterm birth, low birth weight and greater infant weight gain; chronic mucus hypersecretion
- Medications: lack of ICS treatment in patients who had a severe exacerbation
- Exposures: tobacco smoke; noxious chemicals; occupational exposures
- Investigations: low initial FEV_1; sputum or blood eosinophilia

Risk factors for medication side-effects

- Systemic: frequent OCS; long-term, high dose and/or potent ICS; also taking P450 inhibitors
- Local: high dose or potent ICS; poor inhaler technique

BD: bronchodilator; FEV_1: forced expiratory volume in 1 second; ICS: inhaled corticosteroid; OCS: oral corticosteroid; P450 inhibitors: cytochrome P450 inhibitors such as ritonavir, ketoconazole, itraconazole; SABA: short-acting $beta_2$-agonist.

*Based on short-acting $beta_2$ agonist (as-needed ICS-formoterol reliever not included); excludes reliever taken before exercise.

[†]'Independent' risk factors are those that are significant after adjustment for the level of symptom control.

GINA 2022 recommendations for assessing asthma control in two domains: symptom control, and risk factors for poor outcomes. Adapted with permission from Box 2-2 in GINA 2022.[1]

1. Global Initiative for Asthma. *Global Strategy for Asthma Management and Prevention, 2022*, (GINA, 2022). Available at: www.ginasthma.org/reports
